# Autoimmune Encephalitis—A Multifaceted Pathology

**DOI:** 10.3390/biomedicines11082176

**Published:** 2023-08-02

**Authors:** Monica Neațu, Ana Jugurt, Anca Covaliu, Eugenia Irene Davidescu, Bogdan Ovidiu Popescu

**Affiliations:** 1 Department of Clinical Neurosciences, “Carol Davila” University of Medicine and Pharmacy, 050474 Bucharest, Romania; monica.neatu@rez.umfcd.ro (M.N.); ana.jugurt@rez.umfcd.ro (A.J.); anca.covaliu@rez.umfcd.ro (A.C.); bogdan.popescu@umfcd.ro (B.O.P.); 2 Department of Neurology, Colentina Clinical Hospital, 020125 Bucharest, Romania; 3 Department of Cell Biology, Neurosciences and Experimental Myology, “Victor Babeș” National Institute of Pathology, 050096 Bucharest, Romania

**Keywords:** autoimmune encephalitis, encephalitis symptoms, diagnosis autoimmune encephalitis, treatment autoimmune encephalitis

## Abstract

Autoimmune encephalitis is a complex and multifaceted pathology that involves immune-mediated inflammation of the brain. It is characterized by the body’s immune system attacking the brain tissue, leading to a cascade of inflammatory processes. What makes autoimmune encephalitis vast is the wide range of causes, mechanisms, clinical presentations, and diagnostic challenges associated with the condition. The clinical presentations of autoimmune encephalitis are broad and can mimic other neurological disorders, making it a challenging differential diagnosis. This diverse clinical presentation can overlap with other conditions, making it crucial for healthcare professionals to maintain a high level of suspicion for autoimmune encephalitis when evaluating patients. The diagnostic challenges associated with autoimmune encephalitis further contribute to its vastness. Due to the variable nature of the condition, there is no definitive diagnostic test that can confirm autoimmune encephalitis in all cases. In this context, personalized patient management is crucial for achieving favorable outcomes. Each patient’s treatment plan should be tailored to their specific clinical presentation, underlying cause, and immune response. Our objective is to raise awareness about the frequent yet underdiagnosed nature of autoimmune encephalitis by sharing five cases we encountered, along with a brief literature review.

## 1. Introduction

Autoimmune encephalitis is a complex neurological condition characterized by inflammation in the brain due to the immune system attacking healthy brain cells [1,2]. This occurs when the body’s immune system, which is meant to protect against foreign invaders like viruses or bacteria, incorrectly identifies certain components of the brain as foreign and launches an immune response against them [1,2]. This immune response can be triggered by autoantibodies that specifically target various structures in the brain, such as receptors, ionic channels, or supporting proteins on the synaptic surface [1,2]. It can also be triggered by intracellular antigens, like onconeural antigens [1,2]. As a result of this exaggerated immune response, inflammation occurs, leading to significant damage to the structure and function of the brain [1].

Since 2007, there has been a remarkable advancement in our understanding of autoimmune encephalitis, with the discovery of many new antibodies that target neuronal surface antigens and synaptic proteins [1,2,3,4]. These novel antibodies have given rise to various forms of autoimmune encephalitis, such as those caused by autoantibodies targeting GABAB-R, GABAA-R, AMPAR (alpha-amino-3-hydroxy-5-methyl-4-isoxazolepropionic acid receptor), and CASPR2 (contactin-associated protein 2), and the list of such antibodies continues to expand [1,2,3,4,5]. This significant progress has enabled researchers and clinicians to identify more specific subtypes of autoimmune encephalitis, providing valuable insights into the underlying mechanisms of the condition [2,3,4,5]. In 2011, the term “seronegative autoimmune encephalitis” was introduced to highlight a distinct group of immune-responsive encephalitic disorders [1,2,3,4,5]. These cases are characterized by the absence of detectable IgG-class neuronal autoantibodies in the serum, challenging the conventional diagnostic approach [1,2,3,4,5].

It is important to recognize that autoimmune encephalitis is a complex condition, and treatment plans should be customized to each individual based on their specific symptoms, antibody findings, and response to therapy. Early diagnosis and appropriate treatment can lead to substantial improvement in symptoms and a positive prognosis for many individuals with autoimmune encephalitis [1]. However, some cases may present greater challenges in treatment, and long-term neurological complications can arise [2]. Therefore, a personalized approach to treatment and ongoing medical management is essential for individuals affected by this disorder [2,3].

The purpose of this article is to raise awareness about autoimmune encephalitis as a potentially reversible cause of a significant medical emergency. It focuses on the presentation of five cases of primary autoimmune encephalitis treated in the Neurology Clinic of Colentina Clinical Hospital in Bucharest, Romania. The aim is to highlight the variability of this condition from its initial onset to the outcome of the patient.

## 2. Case Presentation

### 2.1. Case 1

In April 2022, a 28-year-old female patient with systemic lupus erythematosus visited our clinic due to a rumination that began four months previous. She described experiencing “songs playing in her mind one after the other”, along with generalized anxiety disorder and depressive disorder. During this period, the patient also had two episodes of auditory hallucinations and persistent optical illusions, seeing “shadows of moving objects”. She reported nightmares and vivid dreams with themes of persecution, such as chases and falls from height. The patient was already receiving at-home treatment with Paroxetine 20 mg once daily.

Upon admission, the clinical evaluation did not reveal any changes, and the neurological examination was mostly normal, except for more vivid osteotendinous reflexes on the left arm, bilateral Hoffman sign, Marinescu-Radovici sign on the right side, Myerson sign present, and bilateral Babinski sign. Further investigations during hospitalization included blood and urine tests, which detected low levels of C3 fraction and normal levels of C4. Additionally, a high titer of anti-LGI1 antibodies (45.4 pmol/L) was found, exceeding the maximum limit of our laboratory (40 pmol/L).

A lumbar puncture was performed to analyze the cerebrospinal fluid, revealing lymphomonocytic pleocytosis, elevated protein levels, and intrathecal IgM synthesis without any alteration of the blood-brain barrier (Table 1). The EEG examination showed frequent, slow, arrhythmic, broad, and generalized wave discharges in the left frontal regions, suggesting a possible lesional substrate. The duration of these discharges ranged from approximately 1 to 4 s, and no epileptiform elements were observed. Native brain MRI did not show any evident lesions, and ophthalmological examination and screening for neoplasms were normal.

Based on the subacute clinical presentation with diverse psychiatric manifestations, CSF and blood analysis, and EEG findings, a diagnosis of autoimmune encephalitis with anti-LGI1 antibodies was established, despite the absence of MRI lesions (note: non-invasive infectious screening was performed). Due to logistic reasons, immunoglobulin therapy was not available, and instead, the patient consented to receive methylprednisolone 1 g/day for 5 days, along with proton pump inhibitors for gastric prophylaxis. This treatment was well-tolerated and led to improvement in some of the psychiatric symptoms, particularly the nocturnal ones. Plasma exchange was postponed due to the mild severity of the presentation. As certain symptoms were paroxysmal and stereotypical, indicating possible ictal events, symptomatic antiepileptic treatment with Valproate 500 mg/day was initiated, considering its stabilizing effect.

In January 2023, the patient returned to the clinic due to persisting visual symptoms and new occurrences. She described parenthesis-like sensations in her upper left arm, especially on the lateral side, which started one week before hospitalization. Additionally, during the same period, she experienced language disorder episodes where she unintentionally switched from speaking in Romanian to English at her workplace. The neurological examination remained unchanged, the MRI showed no lesions, and the EEG indicated improvement compared to the previous evaluation. This was considered a new disease flare-up, leading to the initiation of immunoglobulin therapy at a dose of 1.86 g/kg administered over 5 days (total dose of 100 g) with prophylaxis against anaphylactic reactions and thrombotic events. Pulse therapy with methylprednisolone 1 g/day for 3 days (with associated gastric prophylaxis) was also administered.

In March 2023, following the previous hospitalization, the patient reported a single occurrence characterized by a visual experience of “a green light reflected in the window, without an external source.” However, there were no other indications suggesting a relapse. Another course of immunoglobulin therapy was considered, with a dosage of 1.90 g/kg administered over a period of three days. The patient was advised to undergo QuantiFERON-TB Gold testing to initiate immunosuppressive therapy due to the endemic nature of tuberculosis in our country. This treatment began in April 2023, and the patient is currently receiving Cyclophosphamide 750 mg/m^2^ monthly, which has substantially improved most of her symptoms. No new attacks have been observed since then up until the present time.

### 2.2. Case 2

In July 2022, a 75-year-old male patient with grade II arterial hypertension is assessed following a two-hour episode of amnesia that occurred two days prior to admission. The patient describes the event as being unable to remember the way home from work. Family members also noticed a persistent state of confusion in the hours following the episode, with the patient experiencing difficulty performing everyday tasks such as opening doors and answering the phone. Additionally, the patient suffers from sleep disturbances, increased nighttime sleep duration, and daytime sleepiness.

Upon admission, the general clinical examination reveals no abnormalities. However, the neurological examination shows mild dysexecutive syndrome, reduced language fluency, and occasional inappropriate responses or misunderstandings. EEG tracings indicate changes indicative of diffuse brain damage, but no epileptic events are detected. Blood tests, apart from an elevated CA 19-9 tumor marker level, are within normal limits. Extensive infectious panel results come back negative.

Lumbar puncture reveals clear cerebrospinal fluid with normal pressure, 42 cells (98% mononuclear, 5% polymorphonuclear), increased protein levels, elevated immunoglobulin index, disruption of the blood-brain barrier without intrathecal immunoglobulin synthesis, negative oligoclonal bands, and elevated IgLON5 antibody levels (Table 1).

MRI imaging does not reveal any specific encephalitis-related lesions. However, multiple microangiopathic lesions, as well as carotid and vertebral atheromatosis, are observed, which can be attributed to the patient’s age.

Based on the clinical presentation and CSF analysis results, the diagnosis of autoimmune encephalitis with IgLON5 antibodies is established, leading to the initiation of methylprednisolone therapy at a dosage of 1 g/day for five days. The patient’s condition remains stable during hospitalization, with a single episode of significant but short-lived disorientation followed by complete remission. EEG monitoring after a few days shows a relatively stable appearance, with slight improvement compared to the initial recording.

In September 2022, the patient revisits the clinic with no new manifestations of the pathology but without significant improvement in the initial symptoms. In agreement with the patient and his family, it is decided to perform plasma exchange, consisting of three sessions with a volume of 1.5 times the patient’s weight, alternating with three courses of methylprednisolone at a dosage of 1 g/day. Subsequently, the patient’s condition improves significantly, and he is discharged with a recommendation for QuantiFERON-TB Gold testing to initiate immunosuppressive therapy. Currently, the patient is undergoing treatment with Cyclophosphamide and has not experienced any new disease relapses to date.

### 2.3. Case 3

In December 2022, a 64-year-old male patient with grade II arterial hypertension, type 2 diabetes mellitus, and dyslipidemia visited our clinic due to a memory disorder that started three months previous. The patient was experiencing difficulty remembering recent actions and also reported focal epileptic seizures with secondary generalization, which began two months previous. The patient described experiencing two epileptic events prior to admission.

It is important to note that the patient was already receiving medication for cardiovascular disease, taking perindopril/indapamide 2.5/0.625 mg once daily, and atorvastatin 20 mg once daily for the metabolic disorder. The clinical examination did not reveal any pathological findings, while the neurological examination showed only a fine intentional tremor on the left side of the body, mild impairment of fixation and evocation memory, and slightly slowed verbal rhythm.

During the hospitalization, various laboratory tests were conducted. An analysis of the venous blood showed an increased level of anti-LGI1 antibodies (53.4 pmol/L). A lumbar puncture was performed, which revealed clear cerebrospinal fluid (CSF) with no abnormalities in the blood-brain barrier, no IgM synthesis in the CSF, negative oligoclonal bands, normal immunoglobulin index, and the presence of anti-LGI1 antibodies in high levels (Table 1).

To further investigate the condition, a brain MRI was conducted, which identified a lesion in the right temporal region, suggestive of autoimmune encephalitis. The EEG did not show any pathological changes, but during a 3-h and 30-min video-EEG recording, two epileptic seizures were observed, originating from the right temporal region. The EKG also revealed numerous extrasystoles.

Additionally, a CT scan was performed to screen for possible underlying oncological pathology, but the results and biological markers were within normal limits. During the hospitalization, the patient received a 5-day course of immunoglobulins at a dose of 1.17 g/kg (a total of 100 g). It was also decided to initiate antiepileptic therapy using Levetiracetam at a dosage of 500 mg six times per day.

The patient’s condition showed improvement with the administered treatment, and the symptoms subsided without any further relapses reported up until the present time.

### 2.4. Case 4

A 66-year-old female patient with a history of vitiligo presented to our clinic with complaints of a worsening coordination disorder primarily affecting the left limbs, particularly the legs. She also experienced gait disturbance and diplopia. These symptoms had been progressively worsening for about one week. Notably, the patient had initially reported dizziness and balance disturbances a year ago.

During the initial clinical examination, all findings were within normal limits, except for facial depigmentation and depigmentation on the hands. Neurological examination revealed an unsteady gait, which the patient could only manage for short distances without support. Horizontal nystagmus was observed when she looked to the sides, and vertical nystagmus was elicited during upward and downward gazes. Diplopia was present when looking to the sides. The patient displayed ataxia affecting all four limbs, with more pronounced impairment in the left limbs and mild impairment in the right limbs. Osteotendinous reflexes were brisk and symmetrical, with greater prominence in the upper limbs. Bilateral Babinski sign was present, but there were no motor deficits or sensory disturbances.

Following a comprehensive investigative workup, potential infectious causes such as HIV and Borrelia burgdorferi, as well as autoimmune or tumoral etiologies, were ruled out. Tests including the onconeural antibody blot, anti-neuronal antibodies (which were borderline within the upper limit of positivity), C3-C4 levels, anti-thyroid peroxidase antibodies, and tumor markers were all negative. A lumbar puncture was performed, and cerebrospinal fluid examination revealed a disruption of the blood-brain barrier but no intrathecal antibody synthesis or oligoclonal bands (Table 1). Additionally, a contrast-enhanced thoraco-abdominal-pelvic CT scan showed no significant changes, the EEG did not show any abnormal graph elements, and electromyography results were normal. Brain MRI with contrast revealed mild cerebellar atrophy.

Considering the clinical presentation and investigations, a diagnosis of primary autoimmune encephalitis was considered. Specific antibody testing confirmed the presence of anti-GAD antibodies, further supporting the diagnosis. The patient was treated with pulse therapy using Methylprednisolone, which resulted in symptom improvement. Consequently, it was decided to continue monthly pulse therapy with Methylprednisolone.

### 2.5. Case 5

An 80-year-old male patient with multiple cardiovascular risk factors, including hypertension, dyslipidemia, age, and gender, has a history of recurrent corticosteroid-responsive encephalitis. This condition has caused language disorders, neurocognitive manifestations, and epileptic seizures in 2011, 2012, 2013, and 2016. The patient presented to our clinic with predominant expressive aphasia that started on the morning of admission. Prior to this hospitalization, the patient had been autonomous, without cognitive impairment affecting daily activities, and had not experienced epileptic seizures in recent years. The patient was on outpatient maintenance treatment with Levetiracetam 1500 mg, Acid acetilsalicilicum 75 mg, Rosuvastatin 20 mg, and Pantoprazolum 40 mg.

Upon admission, the clinical examination revealed normal findings. Neurological examination showed an uncooperative patient with predominantly expressive mixed aphasia. The osteotendinous reflexes were brisk and symmetrical. During dynamic evaluation, the patient exhibited drowsiness, axial ataxia, a tendency for retropulsion and lateral deviation while walking, and behavioral abnormalities. There were no definite signs suggestive of epileptic seizures.

The biological workup revealed a mild inflammatory syndrome and a slight deficiency in vitamin B12. Specific autoantibodies related to probable autoimmune central nervous system pathology were measured but yielded negative results. Native brain MRI showed diffuse cortical and internal atrophy and a left parietal micro-arteriovenous malformation. Lumbar puncture results showed normal cellularity, slightly increased protein levels in the cerebrospinal fluid, disruption of the blood-brain barrier, no intrathecal immunoglobulin synthesis, and no evidence of neuro-infections (Table 1). The electroencephalogram was normal. A thoraco-abdominal-pelvic CT scan revealed diffuse pulmonary fibrosis and hepatic lesions consistent with hemangiomas that enhanced slowly. During hospitalization, pulse corticosteroid therapy was initiated with Methylprednisolone 3 g, resulting in favorable progress. Subsequently, a low dose of oral corticosteroid therapy was continued in the following weeks. Considering the vitamin B12 deficiency, substitutive treatment was administered intramuscularly.

In conclusion, the patient’s diagnosis of recurrent corticosteroid-responsive encephalitis, probably limbic autoimmune seronegative, remains valid.

## 3. Discussion

Encephalitis is a complex inflammatory syndrome affecting the brain parenchyma, and it can arise from a range of interconnected immunological predisposing factors, including infections and other various inflammatory processes. [4,5]. In cases of immune-mediated encephalitis, the immune system recognizes specific epitopes as targets for an immune response and reacts accordingly (Table 2) [4]. Systemic inflammatory autoimmune disorders such as systemic lupus erythematosus, acute demyelinating encephalomyelitis (ADEM), post-herpes simplex encephalitis, or paraneoplastic autoimmunity are among the etiological factors associated with immune-mediated encephalitis. However, in some cases, immune-mediated encephalitis can be classified as idiopathic, where no clear primary immunological trigger can be identified [2,5].

### 3.1. Mechanism of Action

Two immune mechanisms have been proposed in autoimmune encephalitis [2,6,7,8,9]. One of the proposed immune mechanisms in autoimmune encephalitis is the presence of autoantibodies targeting synaptic surface structures, such as receptors, ionic channels, or supporting proteins [10]. These antibodies play a role in causing neuronal dysfunction by affecting synaptic transmission in various ways. For example, anti-NMDAR antibodies can cross-link and internalize receptors, leading to altered synaptic transmission. Antibodies like anti-GABAB receptor antibodies may interfere with neurotransmitter binding, while antibodies such as anti-VGKC and anti-LGI1 antibodies can disrupt ion channel function [9,10]. It is important to note that these antibodies do not directly damage neuronal structures or cause significant neuronal apoptosis, so the clinical outcome is generally favorable [2].

The second mechanism in autoimmune encephalitis involves cytotoxic T cell-mediated neuronal destruction [6,7,8,9]. This mechanism is associated with the presence of antibodies targeting cytoplasmic antigens or nuclear onconeural antigens [2,7]. These antibodies are markers for the concurrent pathogenic cytotoxic T cell response, which leads to the destruction of neurons. This immune response is typically associated with a limited response to treatment and can result in a worse neurological outcome due to the rapid destruction of neurons [6,7,8,9,10].

It is widely accepted that circulating neuronal autoantibodies need to penetrate the tightly regulated blood-brain barrier in order to exert their pathogenic effects within the central nervous system (CNS) [11]. However, the specific mechanisms by which these antibodies gain access to the CNS are still not well understood and remain largely unknown [11,12]. Despite ongoing research, the precise mechanisms involved in allowing these autoantibodies to cross the blood-brain barrier have not been fully elucidated. In recent times, the relationship between the brain’s “glymphatic” system and dual lymphatic channels has been discovered [1]. The glymphatic system involves the flow of cerebrospinal fluid (CSF) through periarteriolar and parenchymal extracellular spaces, facilitated by glial cells [12]. This system allows for the passage of small molecules, including central nervous system (CNS) proteins [11,12]. This discovery challenges the long-held belief in the immune privilege status of the CNS, as intracranial antigens can potentially interact with the immune system through these pathways [11,12]. The implications of this relationship on the mechanisms by which circulating neuronal autoantibodies access the CNS are still being investigated [10,11]. Additional possibilities for the access of circulating neuronal autoantibodies to the central nervous system (CNS) include inflammation-induced hyperpermeability of the blood-brain barrier (BBB) [12]. Inflammatory processes can disrupt the integrity of the BBB, allowing exposure and re-exposure of the brain’s self-antigens to the peripheral adaptive immune system [12]. This exposure can trigger the formation of pathogenic neuronal autoantibodies, leading to a breakdown in immune tolerance. The inflammatory response and increased permeability of the BBB create an environment where self-antigens are more accessible to the immune system, potentially contributing to the development of autoimmune responses within the CNS [11,12]. Viral infections are believed to play a role as immune triggers in certain cases of autoimmune encephalitis. These infections can promote the production of cross-reactive autoantibodies that target neuronal self-antigens [11]. Additionally, viral infections can facilitate the entry of these autoantibodies into the central nervous system (CNS) through mechanisms involving proinflammatory cytokines, particularly interleukin-17 produced by Th17 cells [10,11,12]. These cytokines contribute to the proinflammatory response, which can lead to increased permeability of the blood-brain barrier and allow the passage of autoantibodies into the CNS [12,13]. The cross-reactivity between viral antigens and self-antigens, coupled with the proinflammatory environment created by the viral infection, can trigger an autoimmune response targeting the neurons in the CNS [11,12,13]. However, the specific mechanisms and viral triggers involved can vary among different cases of autoimmune encephalitis [11,12,13,14].

### 3.2. Clinical Manifestation

Autoimmune encephalitis presents with a wide range of clinical features that are highly diverse and depend on the specific neuronal antigens targeted by the autoimmune response, as well as the affected regions of the brain. The onset of symptoms is typically acute to subacute, and the progression of the disease is rapid [2].

The neurological and neuropsychiatric symptoms associated with autoimmune encephalitis can vary greatly [15]. They may include psychiatric and behavioral disturbances, such as mood changes, psychosis, or personality alterations [15,16]. As previously stated, in the first case, we observed a patient experiencing auditory hallucinations, persistent optical illusions, and generalized anxiety. Cognitive dysfunction, such as memory deficits or confusion, is also common [15,16,17]. In the second case presented, the patient described an incident where their memory was distorted, leading to an inability to recall the route home from work. Additionally, family members observed a state of confusion in the hours following the episode. In the third case, the patient reported experiencing memory issues related to recent events. Involuntary movements, such as chorea or dystonia, may be observed [15,16,17,18]. Intractable seizures that are resistant to treatment can occur [15,16,17,18,19]. In both the third and fifth cases, the predominant clinical feature observed was the presence of focal epileptic seizures with subsequent generalization. Sleep disturbances, including insomnia or excessive sleepiness, were reported [15,16,17,18,19,20,21,22,23]. As previously mentioned, the first two patients reported experiencing nightmares and vivid dreams characterized by themes of persecution, such as being chased or falling from heights. These individuals also exhibited longer nighttime sleep duration and increased daytime sleepiness. Autonomic instability, manifesting as changes in blood pressure, heart rate, or body temperature regulation, may be present [15,16,17,18,19,20,21,22,23]. Additionally, a decreased level of consciousness ranging from mild impairment to coma can occur [15,16,17,18,19,20,21,22,23]. The recognition of these diverse clinical presentations is crucial for early diagnosis and appropriate management of autoimmune encephalitis [2].

#### 3.2.1. Psychiatric Symptoms

Psychiatric symptoms are indeed common in autoimmune encephalitis and can present as a wide range of manifestations [15]. These symptoms often pose a diagnostic challenge, as they can be mistaken for primary psychiatric disorders [15,16]. The spectrum of psychiatric symptoms includes personality changes, unusual or bizarre behaviors, restlessness or agitation, various anxiety disorders, depressive or manic symptoms, auditory or visual hallucinations, delusions, and catatonia [24]. It is not uncommon for individuals with autoimmune encephalitis to initially be misdiagnosed with primary psychiatric illnesses such as new-onset psychosis, schizoaffective spectrum disorder, or acute mania [24,25]. This is due to the overlap in symptoms and the absence of obvious neurological signs at the early stages of the disease [15,16,24,25]. Treatment of psychiatric symptoms in autoimmune encephalitis can be challenging. Standard antipsychotic medications often do not effectively alleviate the symptoms and may even be associated with a higher incidence of significant side effects, such as neuroleptic malignant syndrome [24,25]. Therefore, it is crucial to consider autoimmune encephalitis as a possible underlying cause when evaluating patients presenting with new-onset psychiatric symptoms, especially in the absence of a clear psychiatric history or an inadequate response to standard psychiatric treatments [15,16]. Collaboration between psychiatrists and neurologists is crucial in the evaluation and management of these cases, ensuring comprehensive care for both neurological and psychiatric aspects of the disease [15,16,24,25].

#### 3.2.2. Seizures

Seizures are a common feature of autoimmune encephalitis and can even be the initial presenting symptom in some cases. These seizures can manifest as either focal (localization-related) or secondarily generalized seizures, including convulsive status epilepticus, which is a prolonged seizure or a series of seizures without full recovery in between episodes [22,26]. Seizures associated with autoimmune encephalitis of immunological etiology tend to have distinct characteristics [2]. They are often frequent, resistant to standard anti-epileptic medications, rapidly progressive, and may occur alongside progressive encephalopathy, which is a progressive deterioration of brain function [2,22]. Certain seizure semiology and electroencephalogram (EEG) patterns are highly suggestive of specific autoimmune encephalitis subtypes [2,26]. For example, facio-brachial dystonic seizures, which are brief motor seizures lasting for 1–3 s and typically involve simultaneous movements of the limbs and face, are highly characteristic of anti-LGI1 encephalitis [22,26]. Recognizing these specific seizure semiology and EEG patterns can aid in the diagnosis and classification of autoimmune encephalitis, allowing for more targeted treatment strategies and improved patient outcomes [2,22,26]. If autoimmune encephalitis is suspected as the underlying cause of seizures, prompt initiation of immunotherapy along with appropriate management of the seizures is crucial to control the disease and prevent further neurological deterioration [22,26].

#### 3.2.3. Movement Disorders

Movement disorders are frequently observed in cases of autoimmune encephalitis, especially in younger individuals [15]. These movement disorders can present with a wide range of phenotypes, including orofacial dyskinesia (abnormal involuntary movements of the face and mouth), paroxysmal dyskinesia (sudden and episodic abnormal movements), chorea (irregular and dance-like movements), dystonia (sustained muscle contractions causing repetitive or twisting movements), myoclonic jerks (brief, shock-like muscle contractions), tremor (rhythmic shaking), cerebellar ataxia (uncoordinated movements due to dysfunction of the cerebellum), and parkinsonian symptoms (such as rigidity, bradykinesia, and resting tremor resembling Parkinson’s disease) [27]. The specific clinical phenotypes of movement disorders seen in autoimmune encephalitis can vary depending on the types of neuronal autoantibodies involved. For example, orofacial dyskinesia is a common feature observed in the mid-late stages of anti-NMDAR encephalitis [5,27].

#### 3.2.4. Cognitive Impairment

Cognitive impairment is a prominent and consistent feature observed in autoimmune encephalitis [16,22,23,24,25,26,27,28,29,30]. It is important to note that even in the presence of striking neuropsychiatric symptoms like psychosis, mild forms of cognitive dysfunction can go unnoticed [28,29,30]. Cognitive impairment in autoimmune encephalitis encompasses a range of deficits, including problems with memory, language, executive functions, sustained attention, and apraxia (difficulties with coordinating voluntary movements) [16,22,30]. Memory dysfunction is often one of the initial signs, characterized by a rapid decline in short-term memory and working memory [30]. Language deficits are also common, involving difficulties in comprehension, decreased verbal output, and, in severe cases, mutism may be observed [28,29,30]. Executive functions, which encompass skills like planning, problem-solving, and decision-making, may be impaired as well [2].

#### 3.2.5. Sleep Disorders

While sleep disorders are not typically listed as diagnostic criteria for autoimmune encephalitis, they are commonly observed in individuals with this condition, although they may sometimes be overlooked [21]. Various sleep disturbances can occur in autoimmune encephalitis, impacting the quality and patterns of sleep [21,31]. One common sleep disturbance is rapid eye movement (REM) sleep behavior disorder, characterized by the loss of normal muscle atonia during REM sleep, leading to the enactment of dreams through motor behaviors [21]. This can result in potentially harmful movements during sleep. Hypersomnia, which refers to excessive sleepiness and prolonged sleep duration, is also frequently reported in autoimmune encephalitis [21,31]. Individuals may experience difficulty staying awake during the day or feel an excessive need to sleep [2]. Sleep fragmentation is another sleep disturbance observed in autoimmune encephalitis [21]. It involves disruptions in the continuity of sleep, with frequent awakenings throughout the night, leading to a non-restorative sleep pattern. Insomnia, characterized by difficulty initiating or maintaining sleep, can also occur [21,31]. Additionally, periodic limb movements during sleep (PLMS) may be present in autoimmune encephalitis [2]. These are repetitive limb movements, often involving the legs, that occur during sleep and can disrupt sleep continuity [21,31].

### 3.3. Diagnosis

The diagnosis of autoimmune encephalitis typically follows the diagnostic criteria established by Graus et al. in 2016 [1]. These criteria emphasize the importance of having a high level of clinical suspicion for autoimmune encephalitis, particularly when other potential causes have been reasonably excluded [16]. In cases where autoimmune encephalitis is suspected, there should be a lower threshold for conducting tests that involve paired cerebrospinal fluid (CSF) and serum samples to confirm the presence of neuronal autoantibodies [1,16].

When evaluating individuals suspected to have autoimmune encephalitis, several diagnostic steps should be considered [2]:

Screening of paired serum and cerebrospinal fluid (CSF) samples: This is crucial for detecting the presence of IgG class antineuronal antibodies, particularly neuronal surface antibodies [1]. Identifying specific antibodies can provide valuable information about the course, management, and prognosis of autoimmune encephalitis [1,32]. In four out of the five cases presented, we successfully identified the specific antibodies responsible for the autoimmune reaction. These included the anti-LGI1 antibody, anti-GAD antibody, and Ig LON5 antibody.

Tumor screening: It is important to screen for underlying malignancies, as certain autoimmune encephalitis conditions have a higher association with tumors [1,2]. Serum and CSF screening for antibodies can be conducted, and whole-body imaging techniques such as a CT scan or PET scan may be performed if initial tumor screening is negative [2,16]. Fortunately, in the cases we presented, tumor screening was performed, and no malignancy was detected.

CSF biomarkers: Analysis of CSF can reveal biomarkers of inflammation or immune activation, including pleocytosis (more than five white blood cells per mm3), oligoclonal bands, and elevated IgG index [1,2,33] (Table 1).

Serum biomarkers: Testing for systemic autoimmune disorders may be relevant in certain clinical cases [1,2].

Brain neuroimaging: Magnetic resonance imaging (MRI) of the brain can be helpful [1]. Unilateral or bilateral hippocampal FLAIR-T2 hyperintensities, with or without transient contrast enhancement, are highly characteristic of limbic encephalitis, both paraneoplastic and non-paraneoplastic [1,2,32].

Brain 18Fluorodeoxyglucose (18F-FDG) positron emission tomography (PET): PET scans can detect metabolic abnormalities in the brain, although they are not specific to the etiology of autoimmune encephalitis [1,2,34].

Electroencephalography (EEG): EEG can reveal abnormalities such as slow-wave activity (focal or diffuse), epileptiform discharges, and electrographic seizures. However, these findings are not specific to autoimmune encephalitis [1,2,35,36]. The variability of this matter is evident as two patients exhibited clinical epileptic seizures, yet we could objectively observe them on EEG in only one of the cases.

Response to immunotherapy trials: A clinically meaningful response to immunotherapy trials in patients with suspected autoantibody-negative psychosis of probable immune origin can provide circumstantial evidence of underlying immune dysregulation or autoimmunity [2,15].

### 3.4. Treatment

While synaptic autoimmune encephalitis can have severe consequences and even be life-threatening, it is important to note that it is often reversible with timely and appropriate treatment using effective immune therapies [1,2]. Early diagnosis and intervention are crucial in achieving a favorable outcome for individuals with this condition [2]. By targeting the underlying immune response and reducing the impact of autoantibodies on synaptic function, immune therapies can help alleviate symptoms, restore neurological function, and improve overall prognosis [1,2,37].

In the management of autoimmune encephalitis, intravenous corticosteroids (1000 mg IV daily for 5 consecutive days), intravenous immunoglobulin (IVIG) (2 g/kg bodyweight IV infusion), and plasma exchange (one session every other day for an average of 5 days) are considered first-line therapies [2,37]. These treatments aim to modulate the immune response and reduce the levels of autoantibodies targeting neuronal structures [1]. It is important not to delay the initiation of these therapies in individuals strongly suspected to have autoimmune encephalitis, even before confirming the presence of neuronal autoantibodies [2]. The decision to start first-line immune therapies is based on the typical clinical presentations and paraclinical findings that suggest an inflammatory process, such as CSF pleocytosis and inflammatory changes affecting mesial temporal lobe structures [1,2,16]. It is crucial to exclude alternative etiologies, such as infections, before considering autoimmune encephalitis as the primary cause [2,33]. The early administration of first-line immune therapies can help prevent further neurological deterioration and improve the chances of a positive treatment response [2]. Once the specific neuronal autoantibodies are identified through confirmatory testing, the treatment approach can be tailored accordingly. However, the timely initiation of treatment should not be delayed while awaiting confirmation of autoantibodies [33]. It is worth noting that the choice and duration of immune therapies may vary depending on individual cases and the specific autoantibodies involved [2,33]. Close monitoring of the patient’s clinical response and regular reassessment of the treatment plan are essential to optimize outcomes in autoimmune encephalitis [2]. In three out of the five cases presented, the patients responded to first-line therapies (Case 3—immunoglobulin, Cases 4 and 5—methylprednisolone). Only two cases required an escalation of therapy, and in our clinic, we opted for cyclophosphamide.

In cases where individuals with autoimmune encephalitis show an inadequate response to first-line immune therapies or experience relapses despite appropriate maintenance therapy, second-line agents are considered [37]. Rituximab (weekly 375 mg/m2 infusions for 4 weeks) or cyclophosphamide (750–800 mg/m2 monthly for 3–6 months) are among the second-line treatment options [2]. Combining rituximab with cyclophosphamide has been found to provide greater therapeutic efficacy in selected severe forms of autoimmune encephalitis [2,37]. Additional medications like tocilizumab (8 mg/kg monthly) and intravenous methotrexate (10 mg weekly for 3–4 weeks) may be effective in treating severe forms of autoimmune encephalitis that are unresponsive to second-line immunotherapies [2,38]. Although there are no consensus guidelines, maintenance therapy for 1-2 years is generally recommended to prevent relapses [38,39,40,41,42,43]. Options for maintenance therapy may include monthly IVIG infusions, high-dose intravenous methylprednisolone pulse therapy, oral prednisone tapering, and the use of steroid-sparing agents such as azathioprine and mycophenolate [2]. Close monitoring of the patient’s response, regular follow-up, and ongoing adjustments to the treatment plan are necessary to optimize outcomes and minimize the risk of relapse [2,38,39,40,41,42,43].

According to a recent expert opinion-based international consensus on the management of concurrent acute psychosis in autoimmune encephalitis, the following recommendations were made [2]:

1. Lower doses and slower titrations: When using atypical or second-generation antipsychotics and benzodiazepines to control agitation and catatonia, it is recommended to start with lower doses and gradually increase the dosage as tolerated. Slower titrations can help minimize the risk of adverse effects and improve overall tolerability [16].
2. Individualized approach: Treatment should be tailored to the specific needs and response of each patient. Close monitoring of symptoms, side effects, and overall clinical status is crucial to guide treatment decisions and adjust medication dosages accordingly [2,16].
3. Collaborative decision-making: A multidisciplinary approach involving psychiatrists, neurologists, and other healthcare professionals is recommended to ensure comprehensive evaluation, accurate diagnosis, and appropriate treatment planning. Collaborative decision-making allows for a more holistic understanding of the patient’s condition and helps optimize treatment outcomes [1,2,16].
4. Regular follow-up: Regular follow-up visits are essential to assess the response to treatment, monitor for any adverse effects, and make necessary adjustments to the medication regimen. Ongoing communication between the patient, their caregivers, and the healthcare team is important for addressing any concerns and optimizing treatment adherence [2].

It is worth noting that these recommendations are based on expert consensus and may be subject to individual variations and specific clinical circumstances [2]. Therefore, it is important for healthcare providers to consider these recommendations in conjunction with their clinical judgment and tailor the management approach to each patient’s unique needs [16].

## 4. Conclusions

As previously mentioned and evident from the cases presented, autoimmune encephalitis is a complex condition that involves molecular intricacies, diverse clinical manifestations, and various treatment approaches. The inherent variability of this pathology makes it a differential diagnosis for numerous other neurological disorders. In our perspective, maintaining a high level of suspicion for diagnosis and implementing personalized patient management form the foundation for achieving favorable patient outcomes.

## Figures and Tables

**Table 1:** CSF findings.

Cases	Oligoclonal Bands	Immunoglobulin Index	Protein Level	Glucose	Number of Cellular Elements
Case 1 anti-LGI1 antibodies	Absent	No alteration in the blood-brain barrier Intrathecal synthesis of IgM 60%	45.70 mg/dL	60 mg/dL	36 elements/mm^3^ 30% neutrophils 70% mononuclear
Case 2 IgLON5 antibodies	Absent	Alteration in the blood-brain barrier No intrathecal synthesis of immunoglobulin	54.7 mg/dL	62.8 mg/dL	42 elements/mm^3^ 98% mononuclear 2% neutrophils
Case 3 anti-LGI1 antibodies	Absent	No alteration in the blood-brain barrier No intrathecal synthesis of immunoglobulin	29.10 mg/dL	74 mg/dL	1 element/mm^3^
Case 4 anti-GAD antibodies	Absent	Alteration in the blood-brain barrier No intrathecal synthesis of immunoglobulin	41.30 mg/dL	78.9 mg/dL	2 elements/mm^3^
Case 5 seronegative	Absent	Alteration in the blood-brain barrier No intrathecal synthesis of immunoglobulin	54.5 mg/dL	67.4 mg/dL	3 elements/mm^3^

**Table 2:** Types of encephalitis [2].

Antigens	Main Presentation	Frequency of Cancer	Type of Malignancy
NMDAR	Panencephalitis, psychiatric manifestations, behavioral disturbances, cognitive impairment, seizures, movement disorders	40%	Ovarian or extraovarian teratomas
LGI1	Limbic encephalitis, short-term memory loss, facial-brachial dystonic seizures, depression, sleep behavior disorders	5–10%	Malignant thymoma, neuroendocrine tumors
CASPR2	Morvan syndrome, delusions, and hallucinations	20%	Malignant thymoma
AMPAR	Limbic encephalitis, encephalopathy, memory loss	65%	SCLC, malignant thymoma
GABAAR	Limbic encephalitis, encephalopathy, intractable epilepsy, behavioral and psychiatric disorders	25%	Malignant thymoma
GABABR	Limbic encephalitis, intractable seizures, short-term memory loss	50%	SCLC
DPPX	Limbic encephalitis, encephalopathy, gastrointestinal symptoms, myoclonus, tremors	<10%	B cell neoplasms
mGluR1	Gait instability, cerebellar ataxia	30%	Hematologic
mGluR5	Ophelia syndrome, psychiatric symptoms, encephalopathy	50%	Hodgkin lymphoma
IgLON5	Sleep disorders	n/k	n/k
DNER (Tr)	Gait instability, cerebellar ataxia	>90%	Hodgkin disease
P/Q type VGCC	Paraneoplastic cerebellar degeneration, gait instability, cerebellar ataxia	>90%	SCLC
GlyR	PERM, stiff-person syndrome, muscle rigidity, spasms, oculomotor disturbance, bulbar symptoms, gait impairment pyramidal signs, cerebellar ataxia	<5%	Malignant thymoma, Hodgkin lymphoma
Amphiphysin	Stiff person syndrome, confusion, memory loss, encephalomyelitis	>90%	Breast cancer, SCLC
Hu (ANNA-1)	Limbic encephalitis or encephalomyelitis, painful sensory neuropathy, cerebellar degeneration, brainstem encephalitis	85%	SCLC, NSCLC, other neuroendocrine tumors, neuroblastoma
Yo (PCA-1)	Cerebellar degeneration	>90%	Ovarian cancer, breast cancer
CV2/CRMP5	Limbic encephalitis, cerebellar ataxia, sensory neuropathy, dementia, chorea, optic neuropathy	>80%	SCLC, thymoma
Ta/Ma2	Limbic encephalitis, short-term memory impairment, sleep disorder, cerebellar and brainstem dysfunction, psychiatric symptoms	>75%	Testicular cancer, NSCLC
SOX-1	Lambert Eaton Myasthenic syndrome, neuropathy, paraneoplastic cerebellar degeneration	>90%	SCLC
GAD	Stiff person syndrome, cerebellar ataxia, intractable seizure	<15%	SCLC, other neuroendocrine tumors, malignant thymoma

AMPA = amino-3-hydroxy-5-methyl-4-isoxazolepropionic acid. ANNA-1 = antineuronal nuclear antibody-type 1. CASPR2 = contactin associated protein 2. CV2/CRMP5 = CV2/Collapsin response mediator protein 5. DNER = Delta/Notch-like epidermal growth factor-related receptor. DPPX = dipeptidyl-peptidase-like protein-6. GABA = amino-butyric-acid. GAD = glutamic acid decarboxylase. GlyR = glycine receptor. GFAP = glial fibrillary acidic protein. Ig LON = immunoglobulin G superfamily containing LSAMP, OBCAM, and Neurotrimin. LGI1= leucine-rich glioma inactivated 1. mGluR = metabotropic glutamate receptor. NMDAR = N-methyl-D-aspartate receptor. PCA-1 = Purkinje cell cytoplasmic antibody-type 1. PERM = progressive encephalomyelitis with rigidity and myoclonus. REM= rapid eye movement. SCLC = small cell lung cancer. VGCC = voltage-gated calcium channel. n/k = not known.

## Data Availability

The raw data supporting the conclusions of this article will be made available by the authors without undue reservation.
